# Graft choice for isolated MPFL reconstruction: gracilis versus semitendinosus

**DOI:** 10.1007/s00590-020-02636-z

**Published:** 2020-02-01

**Authors:** Filippo Migliorini, Andromahi Trivellas, Arne Driessen, Valentin Quack, Markus Tingart, Jörg Eschweiler

**Affiliations:** 1 Department of Orthopaedics, RWTH Aachen University Clinic, Pauwelsstraße 30, 52074 Aachen, Germany; 2 Department of Orthopaedics, David Geffen School of Medicine at UCLA, Suite 755, Los Angeles, CA 90095 USA

**Keywords:** Patellofemoral instability, MPFL reconstruction, Semitendinosus, Gracilis, Tendon, Graft

## Abstract

**Introduction:**

After the first patellar dislocation, most patients report damage of the medio-patellofemoral ligament (MPFL) and surgical reconstruction is required. The purpose of this study is to systematically review current evidence and to clarify the role of the gracilis and semitendinosus tendons as graft for isolated MPFL reconstruction.

**Materials and methods:**

The present systematic review was conducted according to the PRISMA guidelines. The literature search was conducted in October 2019. All clinical trials using the semitendinosus and/or gracilis tendon grafts for isolated MPFL reconstruction in patients with patellofemoral instability were considered for inclusion. Only articles reporting a minimum of 12-month follow-up were considered. The PEDro score was used for the methodological quality assessment.

**Results:**

Data from 1491 procedures were collected. The mean follow-up was 36.12 months. There was comparability among the patient baseline. All the scores of interests (Kujala, Tegner, Lysholm) and range of motion scored better in the semitendinosus group. Moreover, in favour of the semitendinosus group, a statistically significant reduction of the revision surgeries and re-dislocations were evidenced. Apprehension test and persistent instability sensation found any statistical correlations.

**Conclusion:**

Isolated MPFL reconstruction through semitendinosus tendon graft performed better than the gracilis in selected patients suffering from recurrent patellofemoral instability.

## Introduction

Patellofemoral instability is a common cause of complaint in active young patients [1]. After the first dislocation, in about 96% of patients, the medial patellofemoral ligament (MPFL) is significantly damaged and surgical reconstruction can be necessary [2]. The reconstruction of the MPFL reports excellent results and patient satisfaction and is related to a low rate of complications and post-operative failures [3]. In conjunction, the centres performing MPFL reconstruction have doubled in the last decades [4]. For an optimal MPFL reconstruction, the graft choice is of fundamental importance. The graft can be harvested from several tendons, auto- versus allograft or even synthetic graft. However, the most used grafts are the gracilis or semitendinosus tendons [5, 6]. Semitendinosus and gracilis tendons are often preferred grafts for ligament reconstruction because of their intrinsic biomechanical proprieties [7], geometric proprieties [8], availability and low donor-site morbidity [9].

However, there is a lack of clinical studies comparing directly the two tendons, and the best graft for MPFL reconstruction is still unclear. The purpose of this study was to systematically review the current evidence and to investigate which is the best graft between gracilis and semitendinosus tendons for MPFL reconstruction. We focused on the clinical scores, physical examination, further revision surgeries and failures.

## Materials and methods

### Search strategy

This systematic review of the literature was conducted according to the Preferred Reporting Items for Systematic Reviews and Meta-Analyses (PRISMA) [10]. The authors drafted a preliminary protocol to guide the search:

- (P) Population: recurrent patellofemoral instability;
- (I) Intervention: isolated MPFL reconstruction;
- (C) Comparison: semitendinosus versus gracilis tendon graft;
- (O) Outcomes: clinical score and examination, re-operations, failure.

### Literature search

Two independent authors (FM, JE) performed the literature search. In October 2019, the following databases were accessed: Pubmed, Embase, Google Scholar, Scopus. The following keywords were used isolated or in combination: *patellofemoral and/or patellar combined with instability, dislocation, luxation, syndrome combined with MPFL and/or rupture, tear, reconstruction combined with semitendinosus, gracilis, hamstring, tendon, graft, combined with bundle, doubled, single*. The same authors screened the resulting articles. If the title and related abstract matched the topic, the full text was accessed. Furthermore, the bibliographies were screened to find additional articles.

### Eligibility criteria

All studies reporting the outcomes of MPFL reconstruction using the semitendinosus and/or gracilis tendon graft for recurrent patellofemoral instability were considered for inclusion. According to the Oxford Centre of Evidenced-Based Medicine [11], levels of evidence I to IV were included. According to the authors’ language capabilities, articles in English, German, Spanish, Italian and French were included. Only articles reporting data concerning isolated MPFL reconstruction that were included with a minimum of 12 months of follow-up were considered. Only articles reporting quantitative data under the outcomes of interest were considered for inclusion. Techniques, comments, letters, editorials, protocols and guidelines were excluded. Biomechanical, animal and cadaveric studies were also excluded. Articles reporting data on patellofemoral instability after total knee arthroplasty were excluded. Articles reporting data of revision surgeries were also rejected. Articles combining MPFL reconstruction with other proximal or distal alignment were excluded. Disagreements between the authors were debated and solved by a third author (AD).

### Outcomes of interest

Two independent authors (FM, JE) extracted the following data: generalities (author, year, type study), patient demographics (number of knees, mean age), follow-up duration, onset (recurrent or acute), presence of risk factors, patellar and femoral graft fixation. The following outcomes of interest were collected: Kujala Anterior Knee Pain Scale [12], Lysholm Knee Scoring Scale [13], Tegner Activity Scale [14]. In addition, apprehension test, range of motion (ROM), persistent sensation of instability, revisions and re-dislocations were recorded.

### Methodological quality assessment

The PEDro score was used for the methodological quality assessment. Two authors (FM, JE) independently performed the score. This score analysed the papers under several items: clear statement of inclusion and exclusion criteria, allocation, randomization, blinding methods, follow-up duration, point estimates and variability. The final result was a value from 0 (poor quality) to 10 (excellent quality). Values > 6 points were considered acceptable (high quality = 10–8; good quality: 8–6; fair quality = 6–4; poor quality ≤ 3).

### Statistical analysis

For the statistical analysis, we referred to the IBM SPSS Software. The arithmetic mean and standard deviation (SD) for continuous variables were adopted. For binary variables, the odd ratio (OR) effect measure was adopted. The confidence interval was set at 95% in all the binary comparisons. The unpaired *T* test was performed in all the comparisons, with values of *P* < 0.05 considered statistically significant.

## Results

### Search result

**Fig. 1:**
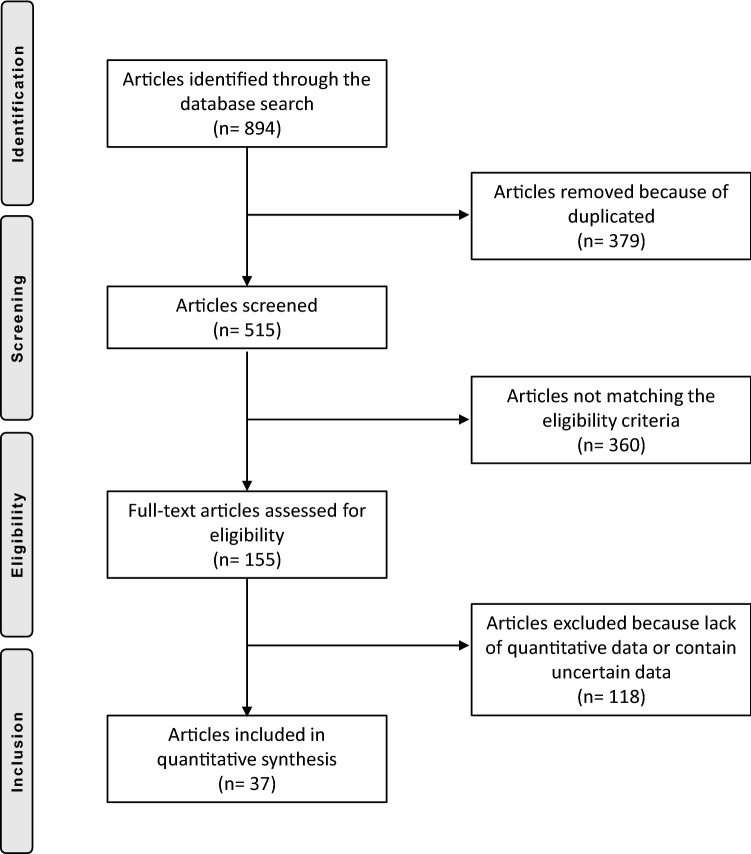
Flow-chart of the literature search

The initial literature search resulted in 894 articles. Of them, 379 were rejected because of duplications. Another 360 were rejected as they did not match the eligibility criteria. A further 107 articles were rejected because no quantitative data under the endpoints of interest were reported. Other 11 articles were rejected due to unreliable data. Ultimately, a total of 37 papers were included in this work, 11 using the gracilis tendon graft and 26 using the semitendinosus tendon graft (Fig. 1).

### Methodological quality assessment

The methodology quality assessment showed several limitations. First, only 8% of the enrolled studies provided a randomization of the samples. Furthermore, no study provided a blinding method of the samples, limiting the quality of the overall results. Strength points included the length of follow-up and the well-designed analysis performed by most of the included papers. Moreover, most articles included a large number of patients in their studies. Consequently, the PEDro score resulted in 6.03 points, attesting to this work a good methodological quality assessment. The results of the PEDro score assigned for each study are shown in Tables 1 and 2.

**Table 1:** Generalities, baseline demographics and related PEDro scores of the included articles reporting data of MPFL reconstruction using a gracilis tendon graft

References	Type of study	PEDro score	Knees (*n*)	Mean age	Mean follow-up	Patellar fixation	Femoral fixation
Astur et al. [15]	RCT	8	30	31.06	60.00	Endobutton	Interference screw
			28	28.32		Anchor	Interference screw
Bitar et al. [16]	PCS	7	56	23.00	19.30	Anchor	Interference screw
Christiansen et al. [17]	PCS	6	32	22.00	22.00	Bone tunnel	Interference screw
Hinterwimmer et al. [18]	RCS	6	19	23.00	16.00	Bone tunnel	Interference screw
Kim et al. [19]	RCS	6	9	24.60	19.30	Soft tissue	Suture anchor
Krishna Kumar et al. [20]	PCS	7	30	18.00	25.00	Endobutton	Interference screw
Lind et al. [21]	PCS	8	24	12.50	39.00	Bone tunnel	Soft tissue
			179	23.00	41.00	Bone tunnel	Interference screw
Lippacher et al. [22]	RCS	7	68	18.30	24.70	Bone tunnel	Interference screw
Thaunat et al. [23]	RCS	5	23	22.00	28.00	Bone tunnel	Suture anchor
Wagner et al. [24]	PCS	6	50	19.00	12.00	Suture anchor	Interference screw
Wang et al. [25]	RCS	6	26	26.30	38.20	Suture anchor	Interference screw

*RCT* randomized clinical trial, *PCS* prospective cohort study, *RCS* retrospective cohort study, *CS* case series

**Table 2:** Results of continuous data

Outcome	Gracilis (*n* = 574)	Semitendinosus (*n* = 917)	*Δ*	*P*
Kujala score	84.95 ± 6.5	89.44 ± 6.1	4.49	0.02
Lysholm score	86.73 ± 4.2	91.17 ± 4.2	4.44	0.04
Tegner score	5.20 ± 0.9	5.85 ± 1.1	0.65	0.2
Range of motion (ROM)	121.55 ± 6.2	134.97 ± 6.8	13.42	0.004

### Patient demographics

In the present study, a total of 1491 MPFL reconstructions, with a mean of 36.12 ± 17.1 months of follow-up, were enrolled. In the gracilis graft group, a total of 574 knees were analysed, with a mean age of 22.39 ± 4.8 years. In the semitendinosus graft group, a total of 917 knees were analysed, with a mean age of 22.68 ± 7.4 years. Between the two groups, there were no significant differences among the samples ages (*P* = 0.5), attesting a good baseline comparability. Patient demographics concerning the gracilis tendon graft group are shown in Table 1. Patient demographics concerning the semitendinosus tendon graft group are shown in Table 2.

### Outcomes of interest

In the gracilis group, the Kujala score showed a mean of 84.95% (SD 6.5), the Lysholm score 86.73% (SD 4.2), and the Tegner 5.20 points (SD 0.9), the ROM 121.55° (SD 6.2). In the semitendinosus group, the Kujala score showed a mean of 89.44% (SD 6.1,), the Lysholm score 91.17% (SD 4.2), and the Tegner 5.85 points (SD 1.1), the ROM 134.97° (SD 6.8). All the scores of interests resulted in favour of the semitendinosus group: Kujala + 4.49% (*P* = 0.02), Lysholm + 4.44% (*P* = 0.04), Tegner + 0.65 points (*P* = 0.2), ROM + 13.42° (*P* = 0.004).

In favour of the semitendinosus group, a reduction of the revision surgeries (OR 0.57; 95% CI 0.2853 to 1.1594; *P* = 0.01) and re-dislocations (OR 0.19; 95% CI 0.0539 to 0.6993; *P* = 0.01) were evidenced. The gracilis reported a not statistically significant reduction of the post-operative apprehension test (OR 1.14; 95% CI 0.6774 to 1.9048; *P* = 0.6), reduction of the persistent instability sensation (OR 1.24; 95% CI 0.4440 to 3.4691; *P* = 0.7). Continuous comparisons are shown in Table 3, while binary in Table 4.

**Table 3:** Generalities, baseline demographics, and related PEDro scores of the included articles reporting data of MPFL reconstruction using a semitendinosus tendon graft

References	Type of study	PEDro score	Knees (*n*)	Mean age	Mean follow-up	Patellar fixation	Femoral fixation
Ahmad et al. [26]	CS	5	20	23.00	31.00	Bone tunnel	Interference screw
Amin et al. [27]	RCS	6	8	22.00	24.00	Bone tunnel	Interference screw
Ballal et al. [28]	PCS	7	20	24.40	12.00	Anchor	Interference screw
Biondi Pinheiro et al. [29]	RCS	7	16	27.10	31.20	Anchor	Interference screw
			21	26.40	34.80	Anchor	Interference screw
Csintalan et al. [30]	CS	5	56	24.30	51.00	Bone tunnel	Interference screw
Deie et al. [31]	RCS	5	31	22.20	39.00	Soft tissue	Bone plug
Gomes et al. [32]	PCS	6	16	26.70	60.00	Bone tunnel	Soft tissue
Gomes et al. [33]	PCS	7	12	19.30	53.00	Bone tunnel	Soft tissue
Goncaives et al. [34]	PCS	6	22	28.60	26.20	Bone tunnel	Interference screw
Han et al. [35]	RCS	6	59	24.30	68.40	Bone tunnel	Interference screw
Howells et al. [36]	PCS	7	155	26.00	16.00	Bone tunnel	Endobutton/Interference screw
Kang et al. [37]	RCT	8	82	28.75	24.00	Soft tissue	Interference screw
Kita et al. [38]	PCS	7	44	25.40	39.00	Bone tunnel	Interference screw
Kumahashi et al. [39]	PCS	6	5	13.60	27.80	Interference screw	Interference screw
Kumahashi et al. [40]	PCS	7	17	22.00	45.00	Interference screw	Interference screw
Lin et al. [41]	RCS	5	18	N/R	35.00	Suture anchor	Interference screw
Ma et al. [42]	RCT	8	32	28.40	40.00	Anchor	Interference screw
Matsushita et al. [43]	RCS	6	21	22.10	44.00	Anchor	Interference screw
			18	23.50	38.00	Anchor	Interference screw
Niu et al. [44]	PCS	7	30	25.00	55.10	Bone tunnel	Interference screw
Nomura et al. [45]	RCS	6	12	24.80	51.00	Bone tunnel	Suture anchor
Panni et al. [46]	CS	5	48	25.00	33.00	Bone tunnel	Interference screw or anchor
Raghuveer et al. [47]	PCS	7	15	29.20	42.00	Bone tunnel	Interference screw or anchor
Sadigursky et al. [48]	PCS	7	31	29.38	12.00	Anchor	Interference screw
Toritsuka et al. [49]	CS	6	20	23.80	30.00	Bone tunnel	Endobutton
Wang et al. [50]	RCS	7	28	29.00	42.00	Anchor	Interference screw
Zhang et al. [51]	PCS	7	60	21.00	96.00	Suture anchor	Interference screw

*RCT* randomized clinical trial, *PCS* prospective cohort study, *RCS* retrospective cohort study, *CS* case series

**Table 4:** Results of binary data

Outcome	OR	95% CI	*P*
Revisions	0.57	0.2853 to 1.1594	0.01
Re-dislocations	0.19	0.0539 to 0.6993	0.01
Apprehension test	1.14	0.6774 to 1.9048	0.06
Persistent instability sensation	1.24	0.4440 to 3.4691	0.07

## Discussion

According to the main findings of this systematic review, we found that the semitendinosus tendon graft performed better overall. Worthy of note was the statistically significant reduction of re-dislocations and revisions rate observed in the semitendinosus group. The endpoints Kujala and Lysholm and range of motion were both statistically significant in favour of the semitendinosus graft group. They showed homogenous values, with poor data variance and small confidence intervals, yielding trustworthy results. Concerning the other analysed endpoints, apprehension test and persistent instability sensation, no statistical differences between the two groups were found.

The MPFL is the most important restraint to patellar lateralization during the first 30° of flexion [52, 53]. Current literature reported no clinical study comparing directly the two grafts. However, the biomechanical proprieties of the grafts have been investigated. The study of Mountney et al. [54] stated that the MPFL ruptured at a mean of 26 ± 7 mm, and the patella dislocated at approximately 50 mm, ensuring an MPFL rupture [54]. Graft choice is of fundamental importance for MPFL reconstruction. Tendon tensile strength and viscoelastic properties are some of the most important mechanical parameters to respect when choosing a graft for a successful ligament reconstruction. The MPFL is a ligament of tissue connecting the tubercle of the adductor on the femur epicondyle to the proximal medial edge of the patella [55]. Although a small structure, this ligament shows a remarkable tensile strength and viscoelasticity. As the native tensile strength of the MPFL is approximately 208 N [54, 56], both gracilis and semitendinosus tendon grafts are far more resistant [5, 6]. In fact, the estimated tensile strength of the semitendinosus and gracilis tendon is 1216 N and 838 N, respectively [57]. The semitendinosus tendon graft has more resistance to traction than the gracilis [58]. Therefore, the semitendinosus represents the most commonly used graft for MPFL reconstruction [59, 60]. However, interest concerning gracilis tendon grafts has recently grown [6]: despite being weaker than the semitendinosus tendon, the gracilis tendon has shown a stiffness value closer to that of the MPFL ligament [6, 61]. Tendon stiffness is the ratio of the force response to the displacement of the myotendinous complex (force change/length change, N/m) [62]. The elastic modulus (slope of the linear portion of the stress–strain curve) of the MPFL has been investigated by two biomechanical studies. Smeets et al. [63] reported the elastic modulus of the MPFL to be 294.6 MPa. Another study conducted by Criscenti et al. [64] stated the elastic modulus of the MPFL to be 116 MPa. The elastic modulus of the semitendinosus and gracilis has been also investigated in biomechanical studies. Smeets et al. [65] reported an elastic modulus of 1036 MPa versus 1458 MPa, respectively. Abramowitch et al. [5] detected an elastic modulus of 484.5 MPa versus 625.5 MPa, while Butler et al. [66] 362.2 MPa versus 612.8 MPa for the semitendinosus and gracilis tendons, respectively. These data confirmed that the semitendinosus provides more resistance to the traction, with reduced elastic modulus compared to both MPFL and gracilis tendon. In selected patients suffering from patellofemoral instability, the resulting lateralizing forces weighing on MPFL are greater than those on a healthy knee. The reconstruction via the semitendinosus tendon graft, being more resistant than MPFL and gracilis, can therefore explain the reduced tendency to re-dislocations and revisions. Therefore, according to the present results, the usage of the semitendinosus tendon graft should be encouraged.

The present systematic review evidenced important limitations. The overall poor quality of the included studies represented an important point of weakness. No study took advantage of blinding methods, and only 8% provided randomization of the samples. Therefore, the data must be interpreted with caution. There is a lack of randomized clinical trials in the current literature, and further high-quality studies are strongly required. The present study performed the analyses regardless of the type of onset of instability, type of patellar and femoral fixation. This represents another limitation of the present work, and further studies are required. Points of strength of this systematic review were the comprehensive nature of the literature search, along with the strict eligibility criteria and rigorous quality assessment. Another point of strength, the methodological assessment of this work, which according to the PEDro score, resulted in a good quality assessment. Furthermore, as confirmed by the Student’s t-test, the study presents an optimal baseline comparability of the samples. All these observations provide an overall reduction of the risk of publication bias, generating feasible results.

## Conclusion

For isolated MPFL reconstruction, the semitendinosus performed better overall. The Kujala and Lysholm scores were both statistically significant in favour of the semitendinosus graft group. The ROM was statistically significant and greater in favour of the semitendinosus graft group. Furthermore, a statistically significant reduction in the failure rate was observed in the semitendinosus group. The gracilis tendon graft group reported a reduction in complication rate, but without statistical significance.
